# Dioscin-Mediated Autophagy Alleviates MPP^+^-Induced Neuronal Degeneration: An In Vitro Parkinson’s Disease Model

**DOI:** 10.3390/molecules27092827

**Published:** 2022-04-29

**Authors:** Shofiul Azam, Md. Ezazul Haque, Duk-Yeon Cho, Joon-Soo Kim, Md. Jakaria, In-Su Kim, Dong-Kug Choi

**Affiliations:** 1 BK21 Program, Department of Applied Life Science & Integrated Bioscience, Graduate School, Konkuk University, Chungju 27478, Korea; shofiul_azam@hotmail.com (S.A.); mdezazulhaque@yahoo.com (M.E.H.); whejrdus10@kku.ac.kr (D.-Y.C.); kgfdkr@gmail.com (J.-S.K.); 2 Melbourne Dementia Research Centre, The Florey Institute of Neuroscience and Mental Health, The University of Melbourne, Parkville, VIC 3052, Australia; md.jakaria@florey.edu.au; 3 Department of Integrated Bioscience & Biotechnology, College of Biomedical and Health Science, Research Institute of Inflammatory Disease (RID), Konkuk University, Chungju 27478, Korea; kis5497@hanmail.net

**Keywords:** MPP^+^, neurotoxicity, autophagy, apoptosis, neuroprotection, Parkinson’s disease

## Abstract

Autophagy is a cellular homeostatic process by which cells degrade and recycle their malfunctioned contents, and impairment in this process could lead to Parkinson’s disease (PD) pathogenesis. Dioscin, a steroidal saponin, has induced autophagy in several cell lines and animal models. The role of dioscin-mediated autophagy in PD remains to be investigated. Therefore, this study aims to investigate the hypothesis that dioscin-regulated autophagy and autophagy-related (ATG) proteins could protect neuronal cells in PD via reducing apoptosis and enhancing neurogenesis. In this study, the 1-methyl-4-phenylpyridinium ion (MPP^+^) was used to induce neurotoxicity and impair autophagic flux in a human neuroblastoma cell line (SH-SY5Y). The result showed that dioscin pre-treatment counters MPP^+^-mediated autophagic flux impairment and alleviates MPP^+^-induced apoptosis by downregulating activated caspase-3 and BCL2 associated X, apoptosis regulator (Bax) expression while increasing B-cell lymphoma 2 (Bcl-2) expression. In addition, dioscin pre-treatment was found to increase neurotrophic factors and tyrosine hydroxylase expression, suggesting that dioscin could ameliorate MPP^+^-induced degeneration in dopaminergic neurons and benefit the PD model. To conclude, we showed dioscin’s neuroprotective activity in neuronal SH-SY5Y cells might be partly related to its autophagy induction and suppression of the mitochondrial apoptosis pathway.

## 1. Introduction

Parkinson’s disease (PD) is one of the most prevalent age-related neurodegenerative diseases characterised by the loss of dopaminergic (DAergic) neurons in the substantia nigra pars compacta (SNpc), the formation of intraneuronal proteinaceous inclusion and Lewy bodies [1,2]. Although the exact etiology of PD is still not clear, evidence suggests that mitochondrial dysfunction and oxidative stress play a major role in PD pathogenesis [3].

1-methyl-4-phenylpyridinium ion (MPP^+^) is an active metabolite form of 1-methyl-4-phenyl-1,2,3,6-tetrahydropyridine (MPTP) [4,5], which is a common neurotoxin used to establish in vitro PD model in human DAergic cell lines such as SH-SY5Y neuroblastoma cells. MPP^+^ translocates through the DAergic neurons by the dopamine transporter (DAT), leading to the inhibition of mitochondrial complex I and ATP synthesis and increases in reactive oxygen species (ROS), which, in turn, causes neuronal degeneration [6].

Autophagy is a cellular homeostatic process by which intracellular components, including damaged organelles, misfolded or aggregated proteins, and intracellular pathogens, degrade into the cytoplasm of the cell [7,8]. In the autophagic process, a double-membrane vesicle, the autophagosome, forms that deliver targeted cellular components to the lysosome and fuse within to form autolysosome, which can degrade and recycle malfunctioned contents of cells. This process has been considered a stress response of cells that promotes cell survival; contrarily, autophagy dysfunction could promote cell death [9], leading to neurodegenerative diseases, cancer and ageing-related diseases [10]. Several studies showed that autophagic dysfunction, such as clearance of sporadic or mutant α-synuclein, is involved in the pathogenesis of PD [2,11,12]. MPP^+^ has been shown to increase the microtubule-associated protein light-chain 3 (LC3)-II/LC3-I expressions, a major marker of autophagy, in SH-SY5Y cells [13], but prolonged exposure to MPP^+^ reduces the LC3 expression, as chronic exposure to MPP^+^ impairs autophagosome [14] by lowering phagophore component ATG5 [15].

**Figure 1:**
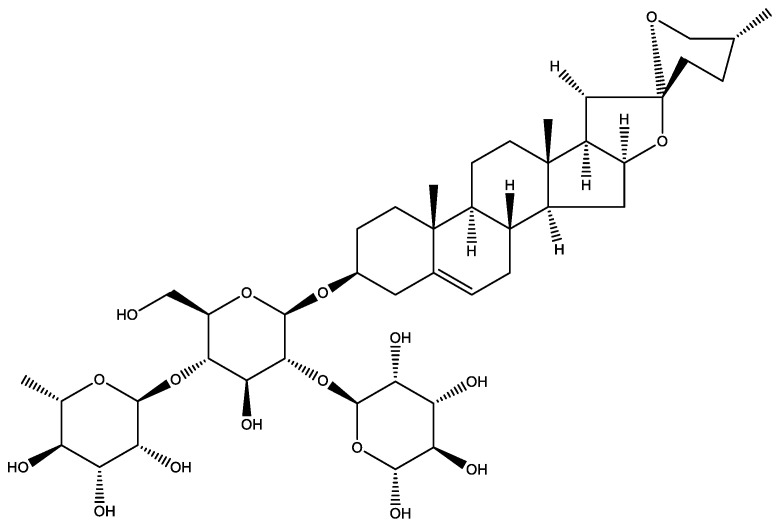
Chemical structure of dioscin; produced by ChemDraw 12.0.2, PerkinElmer Inc., Waltham, MA, USA.

Dioscin is a steroidal saponin (Figure 1), abundant in several medicinal plants, including *Diosocorea nipponica* Makino and *Diosocorea rizhoma*, and it is widely used to synthesise hormonal drugs [16]. Dioscin is also an important raw material of Chinese traditional medicine and is famous for the treatment of aging-related diseases such as AD [17,18] and activities such as anti-inflammation, anti-tumour and anti-allergy [17]. Previous reports showed that dioscin-induced autophagy mitigates cell apoptosis via phosphatidylinositol 3-kinase (PI3K)/protein kinase B (Akt) pathway [16] and alleviates pulmonary inflammation and fibrosis [19]. This steroidal saponin also showed neuroprotective effects in a rat model of cerebral ischemia/reperfusion [20], improved neurogenesis by promoting 5-hydroxytryptamine metabolism [21] and spatial learning memory in ischemic mice [22]. In recent studies, dioscin ameliorated neurodegeneration by reducing oxidative stress and inflammation in rats [18] and inducing autophagy to protect against Aβ_1-42_-induced neurotoxicity in an immortalised mouse hippocampal neuronal precursor cells (HT-22 cells) [17].

Although the molecular mechanism of autophagic involvement in neurogenesis via regulating neurotrophic factors is not fully revealed, recent studies suggest that MPP^+^ precursor MPTP treatment causes partial deficiency of autophagy-related 5 (ATG5) protein [23] in PD animals. Eventually, ATG5 deficiency causes an accumulation of PD proteins in DAergic neurons, leading to apoptosis and downregulation of neurogenesis factors [23]. Further, mitochondrial dysfunction also impairs ATG5-dependent autophagy and causes DAergic cell death via caspase-3 activation [15]. ATG5 is considered a key regulator of autophagic vacuoles and autophagy levels [24]; ATG5 upregulation increases LC3I/II conversion by reducing p62/sequesterosome-1 [23]. We, therefore, speculate that dioscin could prevent ATG5 deficiency, resulting in DAergic neuron transcription factors enhancement and DAergic neurogenesis via increasing expression of neurotrophic factors. We also hypothesise that dioscin-mediated autophagosome restoration may reduce apoptosis, alleviate neuronal survival and protect against damage in a DAergic cell line.

## 2. Results

### 2.1. Dioscin Protects against Neurotoxicity

**Figure 2:**
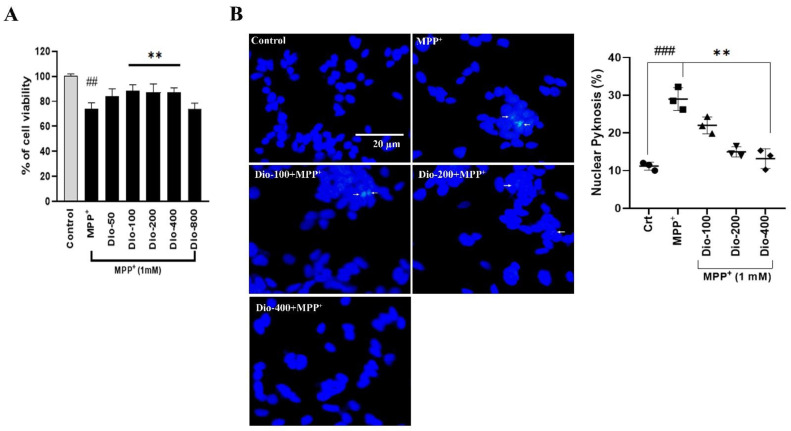
Evaluation of compounds on cell viability. (**A**) dose-dependent cell viability after co-treatment of dioscin and MPP^+^ 1 mM for 24 h; (**B**) fluorescence microscopy of SH-SY5Y cells stained with DAPI, white insides indicating chromatin aggregation (indicated by white arrow); fluorescent images were taken with a widefield fluorescent microscope (Nikon) (scale bar 20 µm); calculations were done by scoring percent of cells with nuclear pyknosis in total stained ones using ImageJ software. Values are mean ± SEM (n = 3/group); ns= not significant; ^#^ *p* < 0.05, ^##^ *p* < 0.01 and ^###^ *p* < 0.001, in comparison to non-treated group; * *p* < 0.05, ** *p* < 0.01 and *** *p* < 0.001, compared to toxin and treatment group.

Firstly, we used different concentrations of MPP^+^ (250–2000 μM) in SH-SY5Y cells and incubated them for 24 h to select the optimal dose of MPP^+^. The cell survivability was dose-dependently reduced by MPP^+^ treatment (Figure S1) that related to previous findings [25,26] and clearly showed that the optimal concentration of MPP^+^ is 1 mM (the dose used for further experiments). Dioscin dose-dependently, except at 800 ng/mL [18], reduced MPP+ activity and increased cell viability; therefore, 100–400 ng/mL of dioscin dose was selected for subsequent study (Figure 2A). To further validate cell viability, prescribed doses of dioscin were checked in LPS-induced BV-2 cells and H2O2-treated PC12 cells (Figures S2A and S2B). Dioscin also alleviated the release of inflammatory cytokines in response to LPS in microglial BV-2 cells (Figure S3). Further, we used the DAPI staining technique to validate dioscin-mediated protection against MPP^+^-induced apoptosis on SH-SY5Y (Figure 2B). MPP^+^ treatment showed significant chromatin fragmentation within the nucleus without changing nuclear phenotype. Pre-treatment of dioscin 200 and 400 ng/mL, but not 100 ng/mL, dose significantly rescued from apoptotic cell death. This result indicates that dioscin improves neuronal cells’ survivability by reducing MPP^+^-induced mitochondrial complex I impairment.

### 2.2. Dioscin Dose-Dependently Downregulates Apoptotic Markers

**Figure 3:**
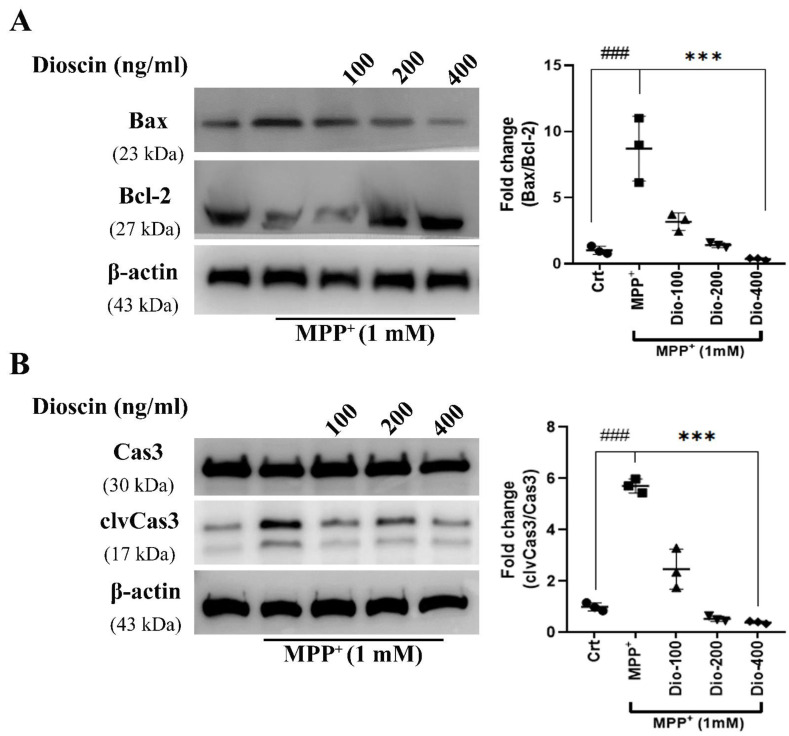
Anti-apoptotic activity of dioscin. Representative data of Bcl-2, Bax (**A**) and Cas3/cleaved caspase-3 (**B**) immunoblots of whole-cell (SH-SY5Y) lysates. Cells were pre-treated with different doses of dioscin followed by MPP^+^ (1 mM) treatment for 24 h (**A**); the ratio of Bax/Bcl-2 is presented here as fold changes of Bax against Bcl-2. The cleaving of clvCas3 from Cas3 is presented as fold changes measured using ImageJ software. Each WB is representative of 3 independent experiments. The bar graphs represent the ratio of respective protein/β-actin normalised against control (non-treated) samples or MPP^+^ or MPP^+^ + treated cells and are mean ± SEM of 3 independent experiments (n = 3). ns = not significant; ^#^ *p* < 0.05, ^##^ *p* < 0.01 and ^###^ *p* < 0.001, in comparison to non-treated group; * *p* < 0.05, ** *p* < 0.01 and *** *p* < 0.001, compared to toxin and treatment group.

In this part, we tested the effect of dioscin pre-treatment on apoptotic biomarkers, B-cell lymphoma 2 (Bcl-2), Bcl-2 associated X protein (Bax), caspase-3, and activated caspase-3 (cleaved caspase-3/clvCas3). Dioscin treatment dose-dependently reduced Bax expression (Figure 3A) but increased Bcl-2 expression. Similarly, dioscin dose-dependently reduced the cleaved caspase-3/ caspase-3 ratio (Figure 3B). Bax plays a major role in MPP^+^-mediated apoptosis, where an increased Bax level reduces anti-apoptotic Bcl-2 and increases the release of mitochondrial cytochrome c. Further, cytochrome c forms complex with Apaf-1, and that activates caspase-3 -mediated apoptosis. This result showed that dioscin protects against MPP^+^-induced toxicity by reducing Bax level, leading to reduced caspase-3 activation.

### 2.3. Dioscin Dose-Dependently Increases TH Cells and Neurotrophic Factors

**Figure 4:**
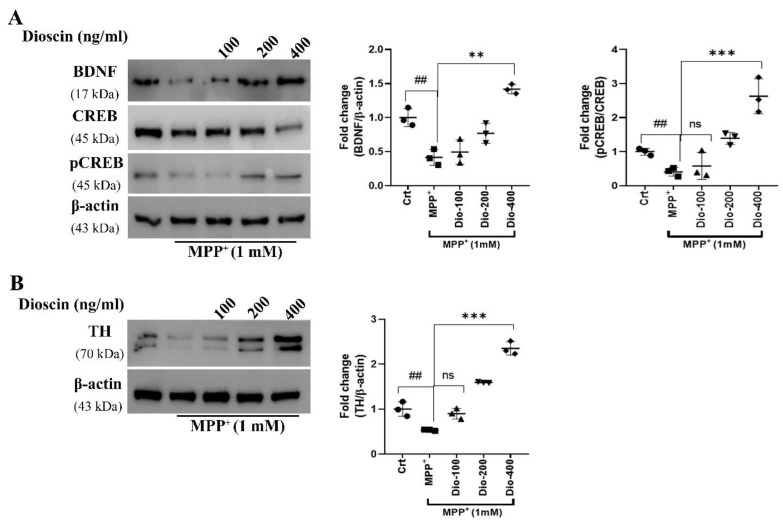
Dioscin improves neurotrophic factors and TH cells. Representative data of TH (first row in the blot image) (**B**), BDNF, CREB and pCREB (**A**) immunoblots of whole-cell (SH-SY5Y) lysates. Cells were pre-treated with different doses of dioscin followed by treatment with MPP^+^ (1 mM) for 24 h (**A**); optical density was measured using ImageJ software. Each WB is representative of 3 independent experiments. The bar graphs represent the ratio of respective protein/β-actin or CREB normalised against control (non-treated) samples or MPP^+^ or MPP^+^ +treated cells and are mean ± SEM of 3 independent experiments (n = 3). ns = not significant; ^#^ *p* < 0.05, ^##^ *p* < 0.01 and ^###^ *p* < 0.001, in comparison to non-treated group; * *p* < 0.05, ** *p* < 0.01 and *** *p* < 0.001, compared to toxin and treatment group.

cAMP response element-binding protein (CREB; phosphor/pCREB) is a transcription factor that could modulate the expression of various other transcription factors like cFos, which in extension regulates brain-derived neurotrophic factor (BDNF) translation. As MPP^+^-induces PD protein aggregation and causes neuronal loss [27], we tested whether or not dioscin-mediated autophagy could rescue neuronal loss via facilitating CREB-dependent BDNF transcription [28]. The result showed a dose-dependent increase in BDNF and pCREB (Figure 4A). The reduction of total CREB at 400 ng/mL dose of dioscin was observed; hence we have also seen significant upregulation of pCREB at that dose. Thereby, the reduction could indicate phosphorylation of CREB and activation of downstream effectors. Time-dependent effect of dioscin on CREB and its downstream effector could draw a mechanistic interpretation of this interesting phenomenon.

Tyrosine hydroxylase (TH) is the key enzyme for DAergic synthesis in the brain. Reduction in TH level causes downregulation of DAergic synthesis, which is key for motor dysfunction syndrome in PD. As the TH level is a positive sign of DAergic synthesis and neurogenesis, our findings support the hypothesis that dioscin promotes DAergic neuronal survival (Figure 4B). In summary, these results suggest that dioscin treatment enhanced BDNF synthesis, possibly via facilitating CREB/cFos/BDNF pathway, while the dioscin pre-treatment increased TH/dopamine levels, an indication of DAergic neurogenesis.

### 2.4. Dioscin Dose-Dependently Rescues Autophagic Function Impaired by MPP^+^

**Figure 5:**
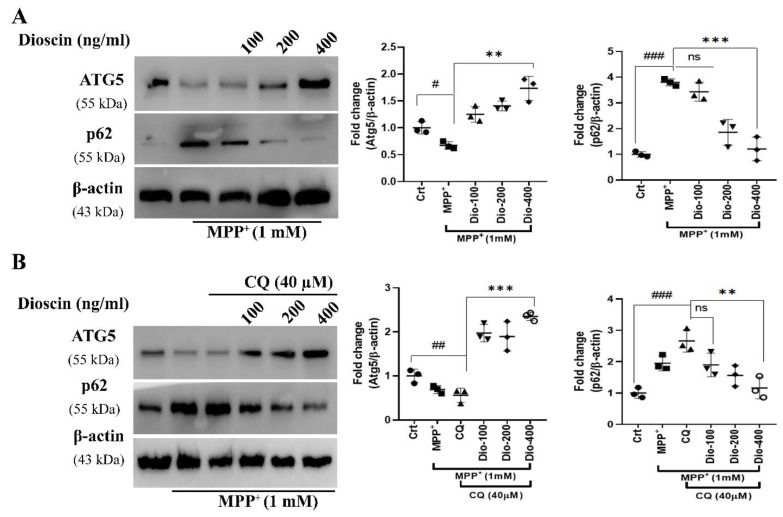
Dioscin dose-dependently activates autophagy. Representative data of ATG5 and p62 immunoblots of whole-cell (SH-SY5Y) lysates. ATG5 and p62 were used as an indicator of sequestosome formation. Cells were pre-treated with different doses of dioscin (**A**) and autophagy inhibitor CQ (40 μM) (**B**), followed by induction of MPP^+^ (1 mM) for 24 h. The optical density was measured using Image J software and presented as fold changes. Each WB is representative of 3 independent experiments. The bar graphs represent the ratio of respective protein/β-actin normalised against control (non-treated) samples or MPP^+^ or MPP^+^ +CQ-treated cells and are means ± SEM of 3 independent experiments (n = 3). ns = not significant; ^#^ *p* < 0.05, ^##^ *p* < 0.01 and ^###^ *p* < 0.001, in comparison to non-treated group; * *p* < 0.05, ** *p* < 0.01 and *** *p* < 0.001, compared to toxin and treatment group.

Besides the Bax-mediated apoptosis, MPP^+^ inhibits autophagosome formation (phagophore phase) by inhibiting ATG5 [15]. We observed that dioscin pre-treatment increased TH/dopamine level significantly (Figure 4B) in the presence of MPP+, so we checked whether it could ameliorate MPP^+^-mediated ATG5 impairment in SH-SY5Y cell lysates (Figure 5A). The ATG5 is a crucial protein marker of autophagic vesicle formation, and a decrease in ATG5 downregulates or inhibits autophagy, suggesting its central role in autophagy [29]. In agreement with a previous study [15], MPP^+^ treatment substantially (^#^ *p* < 0.05) inhibited ATG5, indicating downregulation of autophagic flux formation, which was well supported by the up-regulation of LC3-adaptor protein SQSTM1/p62 (p62) (Figure 5A). p62 is degraded by autophagy, and autophagic impairment results in the aggregation and up-regulation of this protein. Treatment with dioscin dose-dependently increased ATG5 in MPP^+^-induced SH-SY5Y cells, while only 400 ng/mL dose substantially degraded p62 (Figure 5A), indicating amelioration of autophagy. To prove the hypothesis that dioscin affects autophagic flux formation, CQ, a lysosomal inhibitor, was pre-treated 1 h before treating MPP^+^ or MPP^+^ and dioscin (100–400 ng/mL) in SH-SY5Y. The result showed significant (^#^ *p* < 0.05) impairment in MPP^+^ and CQ treated group, and dioscin at the high dose alone increased p62 degradation, but no significant changes were observed in the other two-dose (Figure 5B). A similar potential effect of dioscin treatment was also evident in ATG5 expression (Figure 5B).

### 2.5. Dioscin Dose-Dependently Upregulates Autophagosome Formation

**Figure 6:**
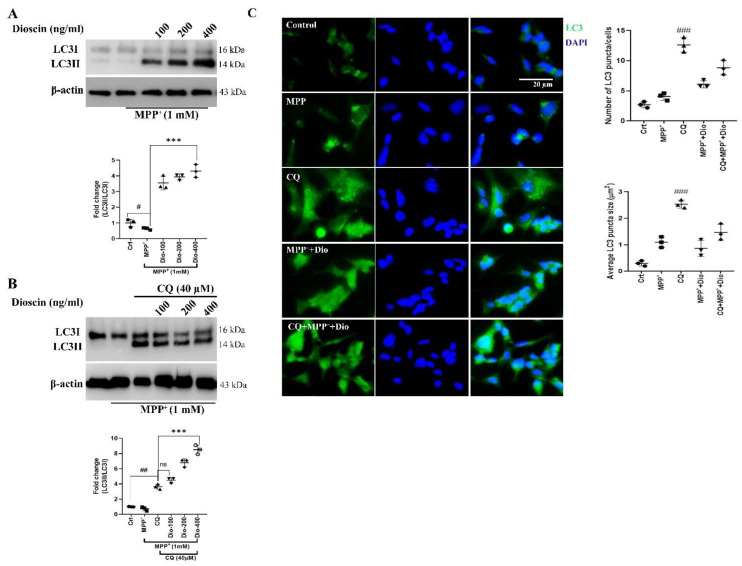
Dioscin dose-dependently activates autophagosome. Representative data of LC3 immunoblots of whole-cell (SH-SY5Y) lysates. Cells were pre-treated with different doses of dioscin (**A**) and autophagy inhibitor CQ (40 μM) (**B**), followed by induction of MPP^+^ (1 mM) for 24 h. The conversion of LC3I into LC3II is presented as fold changes measured using Image J software. Each WB is representative of 3 independent experiments. Data in bar graphs represent the ratio of respective protein/β-actin or LC3I normalised against control (non-treated) samples or MPP^+^ or MPP^+^ + CQ-treated cells and are mean ± SEM of 3 independent experiments (**C**) Immunocytochemistry of autophagic marker LC3 in presence or absence of inhibitor CQ (40 μM) on MPP^+^-treated or untreated SH-SY5Y cells; the images were taken by Nikon microscopy (widefield fluorescent microscope; scale bar 20 µm), and average size and number of LC3 puncta were done by ImageJ software; quantification performed from ≥25 cells. ns = not significant; ^#^ *p* < 0.05, ^##^ *p* < 0.01 and ^###^ *p* < 0.001, in comparison to non-treated group; * *p* < 0.05, ** *p* < 0.01 and *** *p* < 0.001, compared to toxin and treatment group.

Post-treated SH-SY5Y cell lysates were subjected to blot LC3, an autophagosome marker, to investigate the influence of dioscin pre-treatment on the autophagic response. On the activation of autophagy, LC3 is cleaved to form cytosolic LC3I, later converted into LC3II when conjugated to phosphatidylethanolamine [30]. Cells exposed to MPP^+^ for 24 h showed impaired conversion into LC3II, indicating impaired autophagosome formation (Figure 6A). Contrarily, the dioscin treatment group reversed that condition and significantly improved autophagosome formation supported by previous studies [16,19]. Cells were pre-treated with CQ (40 μM) to justify the effect of dioscin on autophagic flux formation and found that CQ increased LC3 level, which is due to CQ capacity of impairing autolysosome degradation; the treatment group at high dose have increased LC3 expression at least 3-fold than MPP^+^ (Figure 6B).

The dioscin-mediated autophagosome-lysosome fusion was further confirmed by fluorescence microscopy (Figure 6C). Both treated and untreated SH-SY5Y cells were stained with an anti-LC3 antibody. Although it is not confirmed what phase of autophagy is blocked by MPP^+^ chronic exposure, we speculated in agreement with previous findings [15] that MPP^+^ might inhibit ATG5-dependent phagophores. So, MPP^+^ treatment showed less fluorescence intensity after anti-LC3 exposure, indicating impairment of autophagosome. As an inhibitor of autophagosome-lysosome, CQ treatment increased LC3 puncta, which indicates inhibition of autophagic fusion. On the other hand, dioscin (400 ng/mL) treatment enhanced autophagic fusion (as seen in Figure 6C) in MPP^+^-treated SH-SY5Y, also reduced LC3 aggregation in CQ-cotreated cells.

## 3. Discussion

While dioscin-treated autophagy activation demonstrates anti-apoptotic and anti-inflammatory properties, it is not clear whether this activation can protect against neurotoxicity in DAergic neurons. Therefore, this study was conducted to examine the role of dioscin-treated activation of autophagy in DAergic neuronal cell death.

Mitochondria play a vital role in cellular energy production, while mitochondrial dysfunction has been shown to contribute to different disease pathogenesis, including PD. MPP^+^ is a commonly used neurotoxin to establish an in vitro PD model, which inhibits mitochondrial electron transport chain complex I [31]. MPP^+^ treatment causes elevation of intracellular ROS levels and impairs mitochondrial function, which, in turn, causes cell death via activation of the apoptotic pathway. Chronic exposure to MPP^+^ deposits into dopamine neurons through a selective uptake by the DAT and induces a PD like syndrome in cell and animal models [32]. Chronic exposure also impairs autophagic flux [14,33], whereas acute exposure can lead to a transient increase of autophagic flux in vitro [33]. Consistent with a previous study [14], this study has found that autophagy reporter protein p62 accumulated and diminished LC3II expression at 24 h of treatment with MPP^+^.

Earlier findings showed that dioscin exerts neuroprotection against toxicity by activating several mechanisms, including inhibition of inflammatory reactions and release of neurotrophic factors [18]. The role of dioscin in autophagy is well studied, but most studies conducted on various tumour cell lines [16] indicated dioscin exerts activities through PI3k/Akt-dependent or mTOR-dependent pathways [16,19] and reported that the autophagy induced by dioscin is usually caused cell death. A recent study showed that dioscin-autophagy might be involved in the neuroprotective activity in Aβ_1-42_ oligomers treated HT-22 cells [17]. The current study supported that result and found that dioscin was found to alleviate MPP^+^-induced autophagic flux impairment.

Mammalian cells impose three primary types of autophagy: macroautophagy, microautophagy and chaperone-mediated autophagy. It is well known that macroautophagy initiates with autophagosome formation, a spherical double-membrane vesicle, which delivers cytoplasmic components into the lysosome. LC3II is a marker of the autophagosome, and the increased expression of LC3II reflects on the formation of the autophagosome. Afterwards, autophagosomes fuse into lysosomes to form autolysosomes and eventually degrade to expel intracellular substrates [8]. Nevertheless, p62 protein starts accumulating when autolysosome degradation is impaired, which remarks the impairment of the autophagy process [33]. This current study investigated the dioscin effect on autophagy markers in the presence of a common autophagy inhibitor, CQ, to ensure dioscin induces autophagy in a mammalian cell line. As an autophagy inhibitor, CQ blocks the delivery of sequestered cargo into lysosomes, impairs autophagic degradation and accumulates autophagosomes [34]. The autophagolysosomal inhibition is indicated by the upregulation of p62 and LC3I-LC3II, which we have seen in presence of CQ. In contrast, dioscin treatment upregulated LC3II, reduced LC3I and p62 expression, indicating degradation of autophagolysosome in presence of lysosomal inhibitor, CQ. In addition, suppressing ATG5 upregulates p62 accumulation and indicates downregulation of autophagy [35]. ATG5 plays a vital role in canonical and non-canonical autophagy and crosstalk with apoptosis [29]. Dioscin pre-treatment was shown to increase ATG5 expression, which was significantly suppressed by the MPP^+^ treatment. This effect of dioscin was substantially reversed in the presence of CQ. CQ has no impact on the formation of autophagosomes, but it inhibits the degradation of autophagosomes [34]. Our results suggest activation of autophagy might play a protective role in MPP^+^-induced activation PD, and dioscin exerted a protective role through inducing autophagy.

It has been shown that PD pathogenesis involves apoptosis and caspase activation that causes DAergic cell degeneration [36,37]. MPP^+^ treatment leads to mitochondrial dysfunction and increases mitochondrial permeability. Eventually, it increases the release of cytochrome c (Cyt C) from mitochondria to plasma, forms apoptosome and leads to activation of caspase-3 [38]. Additionally, Cyt C release is regulated by Bax/Bcl-2 ratio. This study showed that dioscin pre-treatment substantially downregulates the apoptosis pathway by increasing Bcl-2 while decreasing Bax expression, followed by suppressing caspase-3 activation. The inhibition of autophagy reversed the effect. This result indicates that dioscin-induced downregulation of the apoptosis pathway was partially dependent on the autophagy pathway.

Autophagy plays a part in the integrated pro-survival signalling, including AKT/CREB and defects in autophagy lead cells toward necrotic death [39]. In this study, our prime focus was on cell survival activity exerted by dioscin, precisely CREB/BDNF expression. Since activation of CREB by the phosphorylation, pCREB binds to the cAMP response element (CRE) site and triggers a gene transcription that includes cFos [28,40,41,42]. Evidence indicates that cFos is a regulator of BDNF expression and is responsible for the survival and differentiation of neurons [43]. However, BDNF itself can induce cFos transcription in a feedforward cascade [44,45]. MPP^+^ treatment impairs Ca^2+^ influx, leading to Akt/mTOR pathway inactivation in SH-SY5Y cells that causes downregulation of BDNF and pCREB and degenerates TH-positive neurons [42,46,47]. Disocin treatment ameliorated cells’ survivability via increasing BDNF, pCREB and TH expression in the MPP^+^-treated cellular PD model.

## 4. Conclusions

To conclude, dioscin ameliorated autophagic flux impairment that might be involved in its neuroprotective effect against MPP^+^-induced cell apoptosis in the SH-SY5Y human neuroblastoma cells line. Further investigation revealed that dioscin upregulates neurotrophic factors and dopamine precursor TH. The autophagy pathway plays a significant role in dioscin-mediated neuroprotection on SH-SY5Y neuroblastoma cells, but several limitations of this study need to be overcome by future studies, including in MPTP-intoxicated, trans-genic and autophagy impaired (ATG5^-/-^) animals. In addition to these validations, pharmacokinetic properties of dioscin, such as bioavailability, blood-brain barrier permeation and therapeutic window, need to be studied.

## 5. Methods and Materials

### 5.1. Chemicals

Dioscin (≥98% purity) was purchased from Chem Faces (Wuhan, Hubei 430056, China), 1-methyl-4-phenylpyridinium (MPP^+^), chloroquine diphosphate (CQ; C6628), dimethyl sulfoxide (DMSO) and 3-(3,4-dimehylthiazol-2-yl)-2,5-diphenyltetrazolium bromide (MTT) were obtained from Sigma-Aldrich (St. Louis, MO, USA). Foetal bovine serum (FBS) (#16000-442; Gibco, Brooklyn, NY, USA), phosphate-buffered saline (PBS) and Dulbecco’s modified Eagle’s medium (DMEM/F12) were purchased from Gibco-BRL Technologies (Gaithersburg, MD, USA). RIPA buffer (10x) was purchased from Millipore (Milford, MA, USA), and protease and phosphatase inhibitors were obtained from Roche (Indianapolis, IN, USA). All other chemicals utilised in this research were of analytical grade and were purchased otherwise noted Sigma-Aldrich.

### 5.2. Cell Culture and Treatment

Human neuroblastoma SH-SY5Y cells were obtained from the American Type Culture Collection (ATCC; Manassas, VA, USA), as mentioned earlier [48]. The neuroblastoma cells were cultured in DMEM/F12 (1:1) supplemented with 100 U/mL penicillin/streptomycin and 10% (*v*/*v*) inactivated FBS in an incubator, condition maintained at 37 °C temperature and 5% CO_2_. The cells were trypsinised (0.05% trypsin-EDTA) after reaching 80–90% confluence for sub-culture, and the media was replaced every 2 days interval. Each experiment was conducted at least three times from three consecutive passages for statistical analysis.

The SH-SY5Y cells were exposed to MPP^+^ (1 mM) for 24 h to establish the PD model. During the experiment, different doses of dioscin (100–400 ng/mL) were added to SH-SY5Y cells 2 h before the addition of MPP^+^ (1 mM), and the cells were maintained at 37 °C with 5% CO_2_ conditioned incubator. For the study of autophagy, CQ (40 µM) were incubated 1 h before MPP^+^ treatment.

### 5.3. Cell Viability Assay

The SH-SY5Y cells were seeded at 2.5 × 10^5^ cells/mL in a 96-well plate to assess viability using the previously described MTT assay [48,49]. Briefly, cells were incubated for 24 h with a culture medium before treating respective chemicals at the prescribed dose. Cell culture medium was then replaced with medium containing MPP^+^ (0.25–2 mM) or MPP^+^ and dioscin (50–800 ng/mL), MPP^+^ and dioscin for another 24 h. Later, 20 µL of MTT (5 mg/mL) was added to each well, and the plate was incubated for an additional 3 h at 37 °C and 5% CO_2_. The medium was then carefully removed using a suction pump, and 200 µL of DMSO was added to each well and shaken for 30 mins to dissolve formazan crystals. The absorption was taken at 552 nm using a microplate reader (Sunrise^TM^, Tecan Trading AG, Switzerland).

### 5.4. Western Blot Analysis

The cells were washed twice with cold PBS and subjected to lyse using lysis buffer (1× RIPA lysis buffer containing protease- and phosphatase inhibitor (1:1) cocktail). Whole mixtures were centrifuged at 14,000 rpm at 4 °C for 15 min, and the supernatants were collected carefully without disturbing pellets. The total protein obtained from cell lysates was quantified using DC^TM^ protein assay kit (Bio-Rad, Hercules, CA, USA) according to manufacturer instruction. Equal amounts of protein (20 µg) were separated electrophoretically using 8~15% sodium dodecyl sulphate-polyacrylamide electrophoresis (SDS-PAGE) gel and then transferred onto polyvinylidene-difluoride (PVDF) membranes (Millipore, Bedford, MA, USA). The membranes were incubated at room temperature for 1 h with 3% bovine serum albumin in Tris-buffered saline (containing 0.1% Tween 20 buffer) to prevent nonspecific binding. Subsequently, the blots were incubated overnight at 4 °C with specific primary antibodies, including anti-Caspase3 (#H-277; santa cruz biotechnology), anti-clvCaspase3 (#9661; cell signaling technology), anti-pCREB (#87G3; cell signaling technology), anti-TH (#ab112; abcam), anti-BDNF (#ab226843; abcam), anti-SQSTM1/p62 (#ab109012; abcam), anti-ATG5 (#ab108327; abcam), anti-Bax (#*p*-19; santa cruz biotechnology), anti-Bcl-2 (#N-19; santa cruz biotechnology) and anti-LC3 (#ab192890; abcam) at 1:1000, and anti-β-actin (#C4; santa cruz biotechnology) at 1:5000 concentration. Next, each blot was incubated at room temperature with either anti-mouse or anti-rabbit (1:10,000) secondary antibody. The blots were visualised with an enhanced chemiluminescence detection system (LAS 500; GE Healthcare Bio-Sciences AB, Sweden) as per the recommended protocol.

### 5.5. Immunofluorescence

The immunocytochemistry was performed as described previously [50]. Briefly, SH-SY5Y cells were grown to a confluency of 70–80%, washed with cold PBS once, followed by fixation with 4% cold PFA for 15 min at room temperature. After discarding PFA, cells were washed (twice) and permeabilised with 0.1% Triton X-100 for 10 min at room temperature prior to blocking with 5%BSA (in 0.1%PBS-Tween 20) for 1 h at room temperature. Cells were then washed and incubated with primary antibody (anti-LC3; #ab192890; abcam) at 2 µg/mL for overnight at 4 °C. The cells were counterstained with chicken-anti-rabbit (CAR-594; A21201 Invitrogen) at 1:400 in 5%BSA for 1 h at room temperature before staining with DAPI (2 µg/mL). Images were immediately captured using Nikon Eclipse Ts2R fluorescence microscopy system and were processed by NIS-Elements BR-2.01.00 software (came with the instrument). Cells were stained with DAPI (2 µg/mL) for apoptosis investigation after fixation and permeabilisation.

### 5.6. Statistical Analysis

Statistical analyses were performed using GraphPad Prism (version-8.0.1; La Jolla, CA, USA) software. Data represented as means ± SEM (standard error mean) of three independent experiments. All data were analysed using one-way ANOVA followed by Sidak’s multiple comparisons to determine the significance; the *p*-value was set <0.05 to be considered statistically significant.

## Data Availability

The datasets generated and/or analysed during current study are publicly available.

## Supplementary Materials

The following supporting information can be downloaded at: https://www.mdpi.com/article/10.3390/molecules27092827/s1, Figure S1: Dose-dependent toxicity of MPP+ in SH-SY5Y cells at 24 h; Figure S2A: Cell viability and NO release from LPS-treated BV-2 cells after dioscin treatment at 24 h; Figure S2B: Cell viability of H2O2-treated PC12 cells after dioscin treatment at 24 h; Figure S3: Inflammatory biomarkers regulated by dioscin against LPS-induced in BV-2 microglial cells at 24 h; Figure S4: Dose-dependent autophagic impairment by CQ in SH-SY5Y cells at 24 h. The original blots (Figures 3’6) can be found in Supplementary Materials.

## Sample Availability

Samples of the compounds are not available from the authors.

## Abbreviations

Parkinson’s disease: PD; Dopaminergic: DAergic; Substantia nigra pars compacta: SNpc; Reactive oxygen species: ROS; Dopamine transporter: DAT; 1-methyl-4-phenyl-1,2,3,6-tetrahydropyridine: MPTP; 1-Methyl-4-phenylpyridinium ion: MPP^+^; Microtubule-associated protein light-chain 3: LC3; Sequestosome 1: SQSTM1/p62; Autophagy-related protein: Atg5; B-cell lymphoma 2: Bcl-2; Bcl-2 associated X protein: Bax; Activated Caspase-3: clvCas-3; cAMP response element: CRE; Cytochrome c: Cyt C; Tyrosine hydroxylase: TH; Brain-derived neurotrophic factor: BDNF; Phosphor-cAMP response element-binding protein: pCREB.
