# Supplementary material for: Dioscin-Mediated Autophagy Alleviates MPP+-Induced Neuronal Degeneration: An In Vitro Parkinson’s Disease Model
Source: Molecules. 2022 Apr 29;27(9):2827. doi: 10.3390/molecules27092827 (PMC9104838; doi:10.3390/molecules27092827)

Dioscin-mediated autophagy alleviates MPP+-induced neuronal degeneration: an in *in vitro* Parkinson's disease model

Shofiul Azam [1], Md. Ezazul Haque [1], Duk-Yeon Cho [1], JoonSoo Kim [1], Md. Jakaria [3], In-Su Kim [2], Dong-Kug Choi [1, 2*]

[1] Department of Applied Life Science & Integrated Bioscience, Graduate School, BK21 Program, Konkuk University, Chungju 27478, Korea; shofiul_azam@hotmail.com (SA); mdezazulhaque@yahoo.com (MEH); whejrdus10@kku.ac.kr (DYC); kgfdkr@gmail.com (JSK)

[2] Department of Integrated Bioscience & Biotechnology, College of Biomedical and Health Science, Research Institute of Inflammatory Disease (RID), Konkuk University, Chungju 27478, Korea; kis5497@hanmail.net (ISK)

[3] Melbourne Dementia Research Centre, The Florey Institute of Neuroscience and Mental Health, The University of Melbourne, Parkville, VIC, 3052, Australia; md.jakaria@florey.edu.au (MJ)

*Correspondence: choidk@kku.ac.kr; Tel.: +82-43-840-3610; Fax: +82-43-840-3872

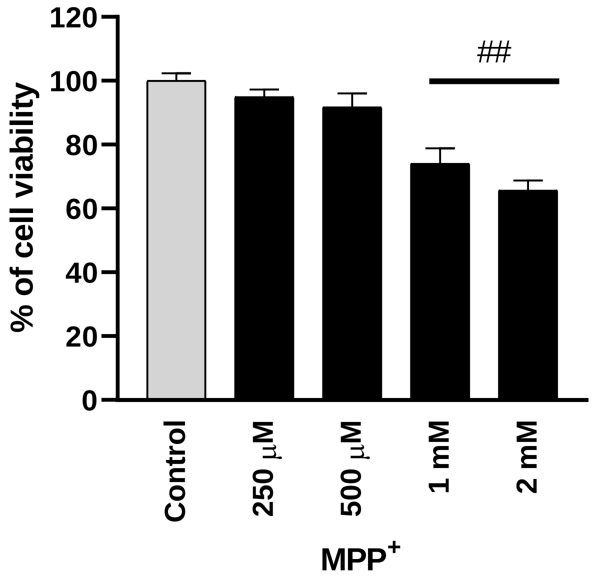

Figure S1: Dose-dependent toxicity of MPP^+ in SH-SY5Y cells at 24 h. For dose determination of MPP^+, different doses of MPP^+ was treated to SH-SY5Y cells for 24 h; n=3; ##p<0.001 compared between non-treated (control) vs treatment (MPP^+), statistical analysis was done using one-way ANOVA.

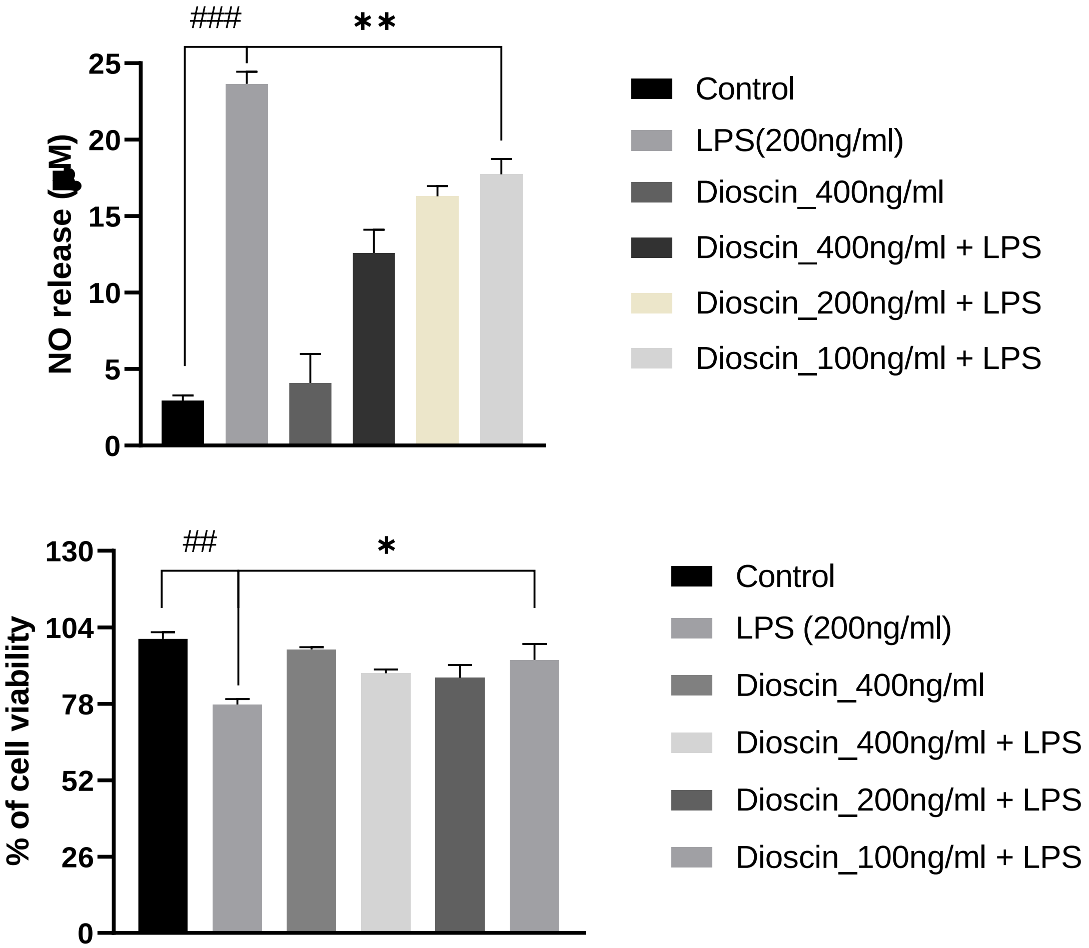

Figure S2A: Cell viability and NO release from LPS-treated BV-2 cells after dioscin treatment at 24 h. BV-2 cells were co-treated (LPS and dioscin) or non-treated for 24 h, LPS-activated microglial cells released NO and decreased viability. n=3; ###p<0.001, ##p<0.01 compared between non-treated (control) vs LPS, and **p<0.01, *p<0.05 compared between dioscin vs LPS, statistical analysis was done using one-way ANOVA.

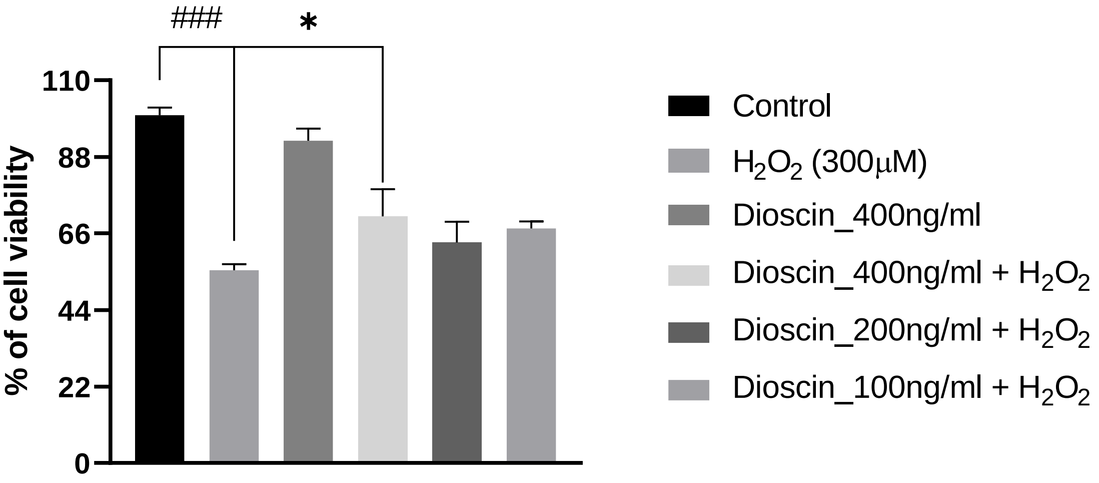

Figure S2B: Cell viability of H_2O_2-treated PC12 cells after Disocin treatment at 24 h. For the cell viability, PC12 cells were either co-treated (H_2O_2 and dioscin) or non-treated for 24 h; n=3, ###p<0.001 compared between control vs H_2O_2 and *p<0.05 compared between H_2O_2 vs dioscin, statistical analysis was done by one-way ANOVA.

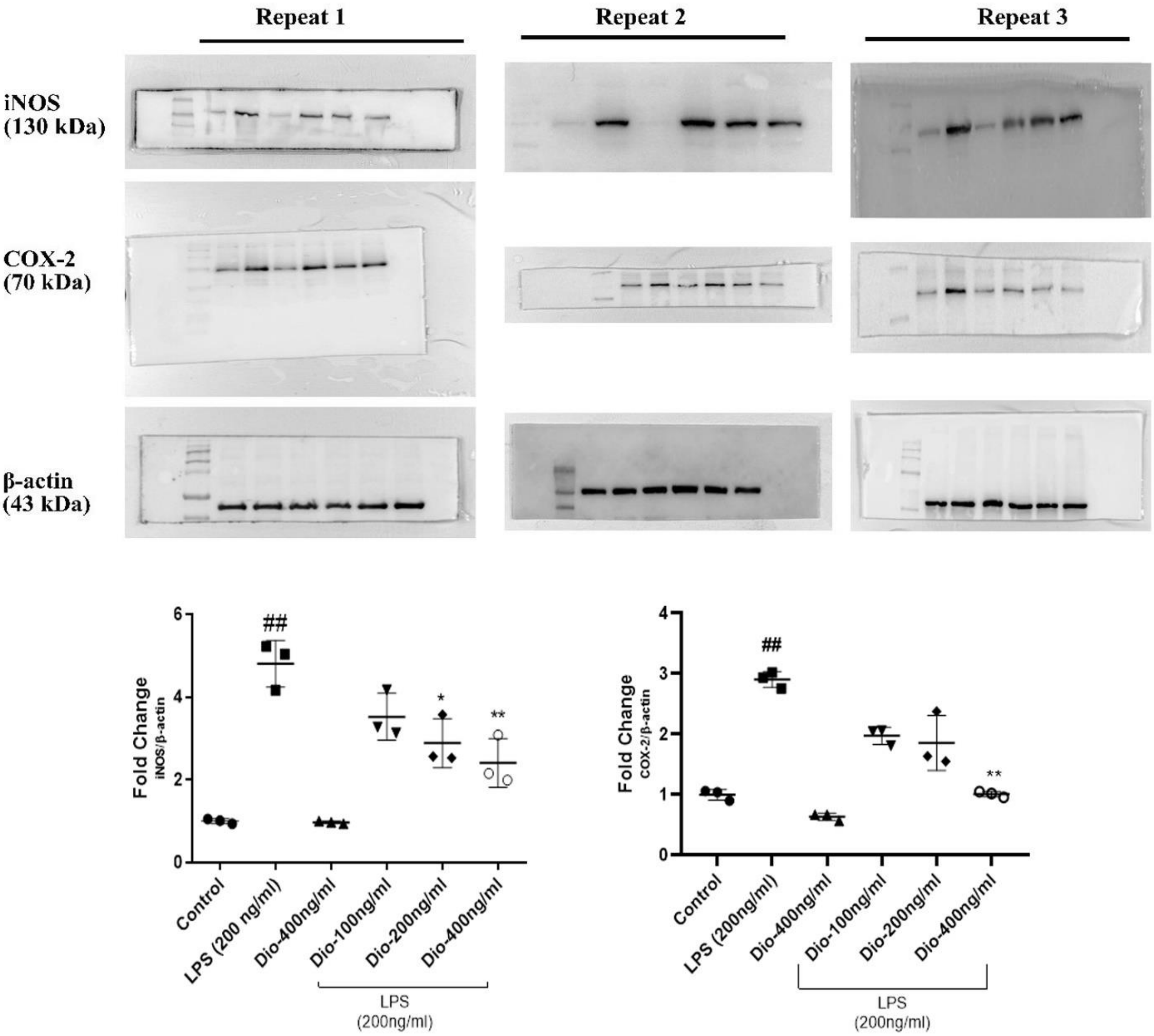

Figure S3: Inflammatory biomarkers regulated by Dioscin against LPS-induced in BV-2 microglial cells at 24 h. BV-2 cells were pre-treated (dioscin) for 2 h prior to incubation with LPS (200 ng/ml) or non-treated for 24 h, LPS-activated microglial cells increasing iNOS and COX-2 expression. n=3; ###$p<0.001$, ##$p<0.01$ compared between non-treated (control) vs LPS, and **$p<0.01$, *$p<0.05$ compared between dioscin vs LPS, statistical analysis was done using one-way ANOVA.

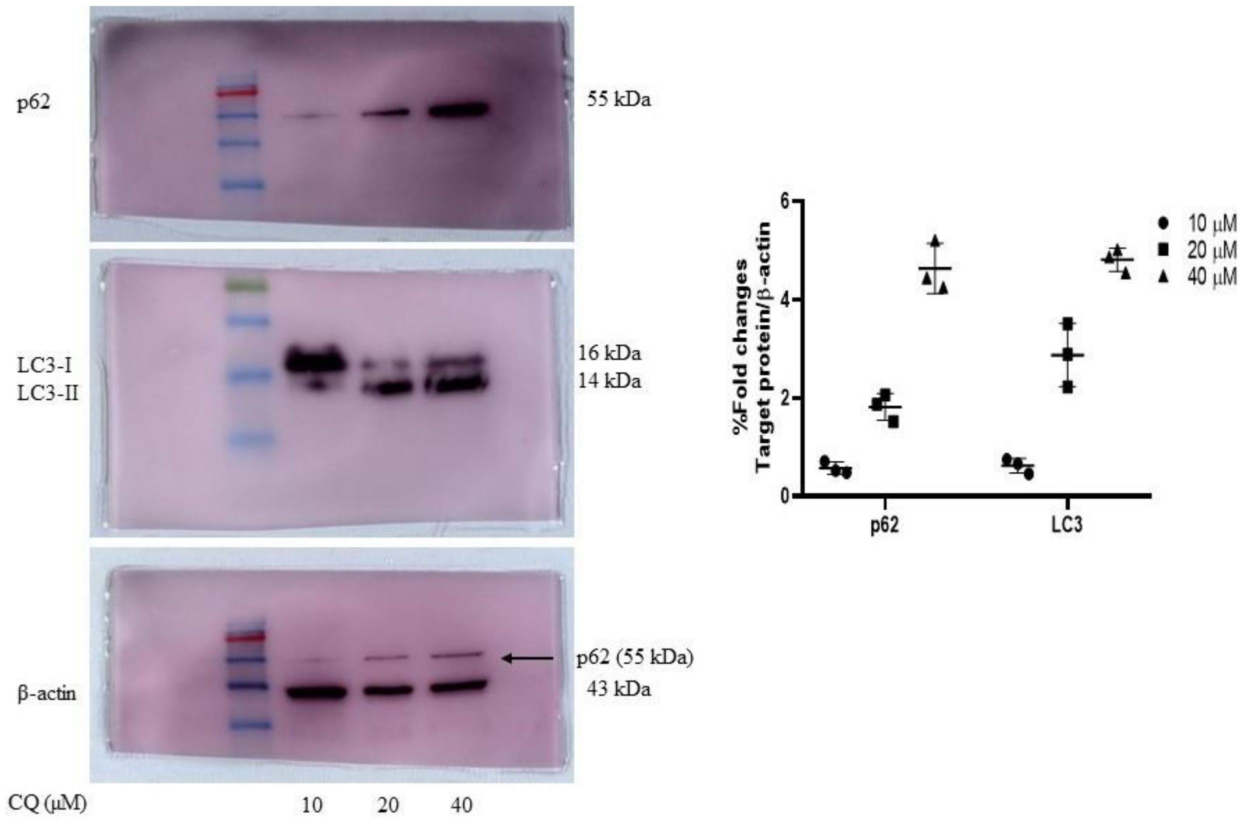

Figure S4: Dose-dependent autophagic impairment by CQ in SH-SY5Y cells at 24 h. For dose determination of CQ, SH-SY5Y cells were treated with different doses of CQ for 24 h and proteins were tested via WB for LC3 and p62 expression; n=3, expression of target proteins were neutralized against β-actin, no statistical analysis performed.

Original blots below are containing proteins per lane as mentioned in the main manuscript.

Sample full blots of cropped images

Figure 3.

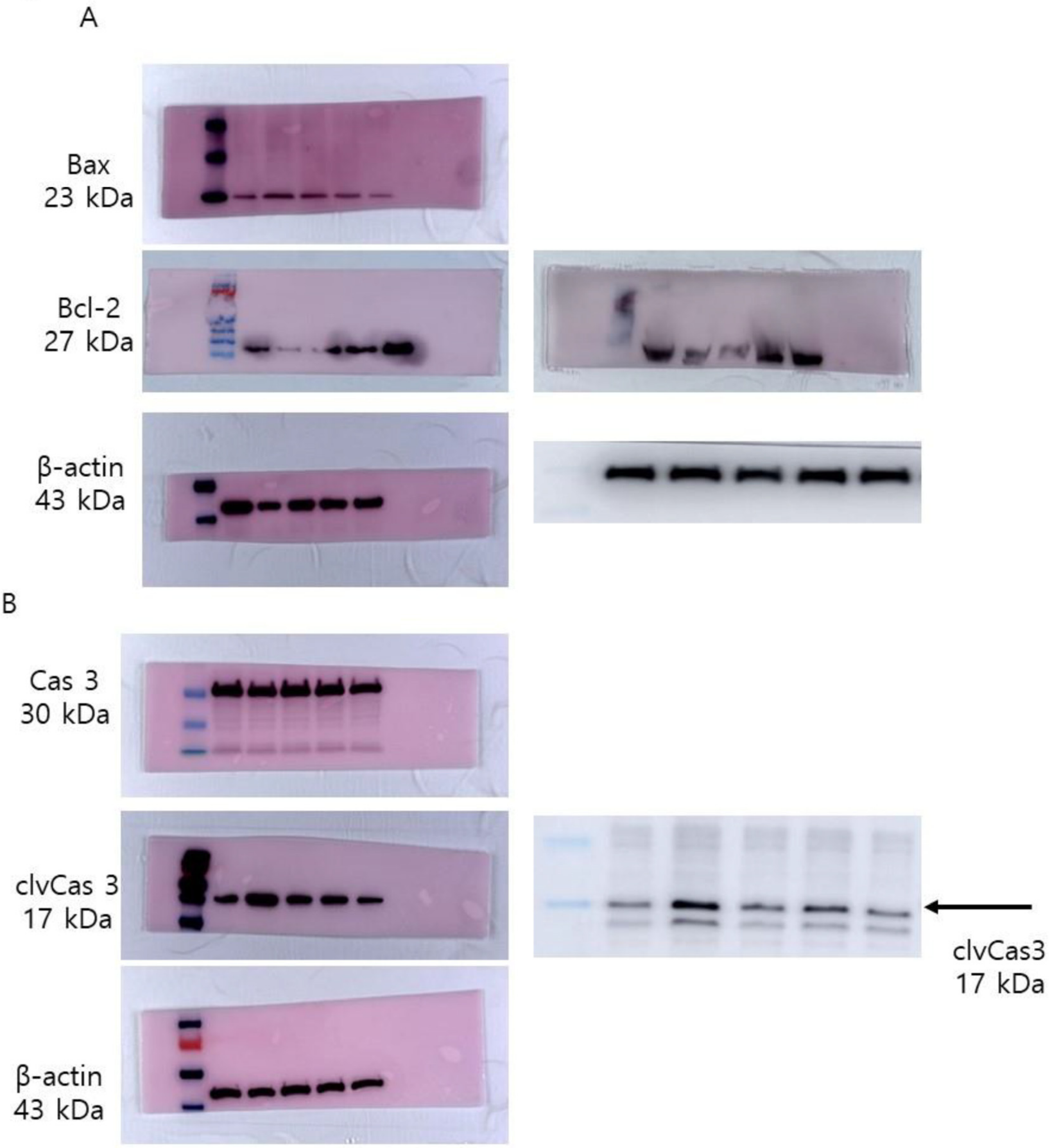

Sample full blots of Cas3/clvCas3

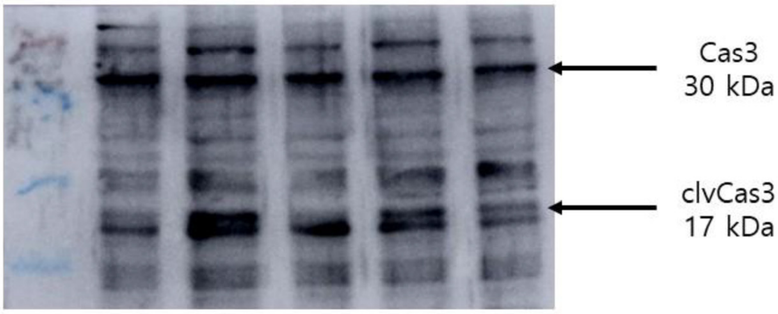

Sample full blots of cropped images

Figure 4.

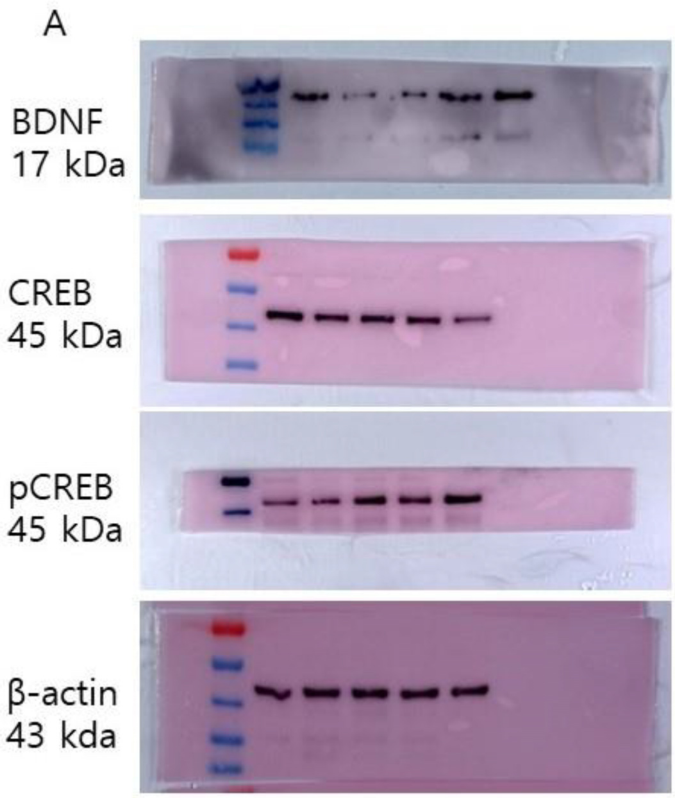

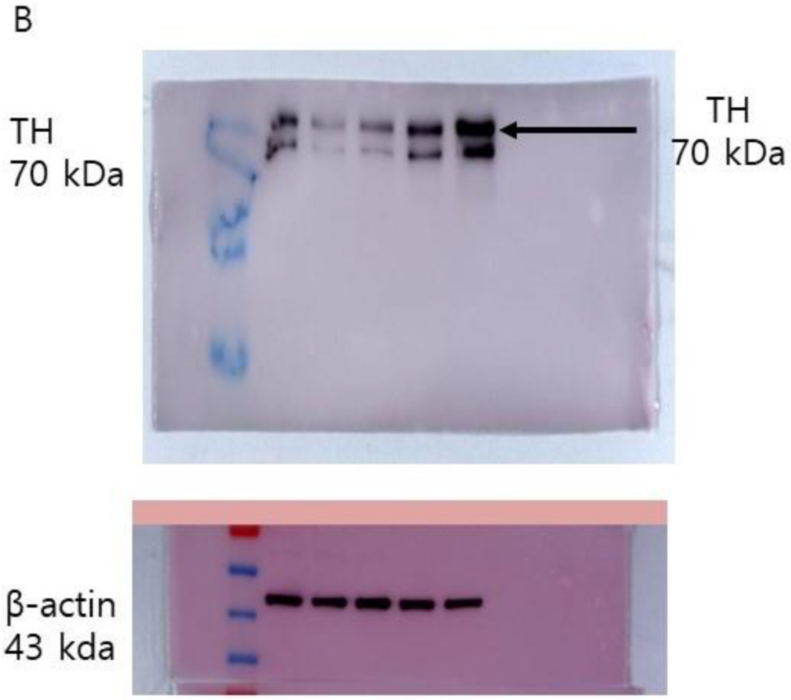

Sample full blots of cropped images

Figure 5.

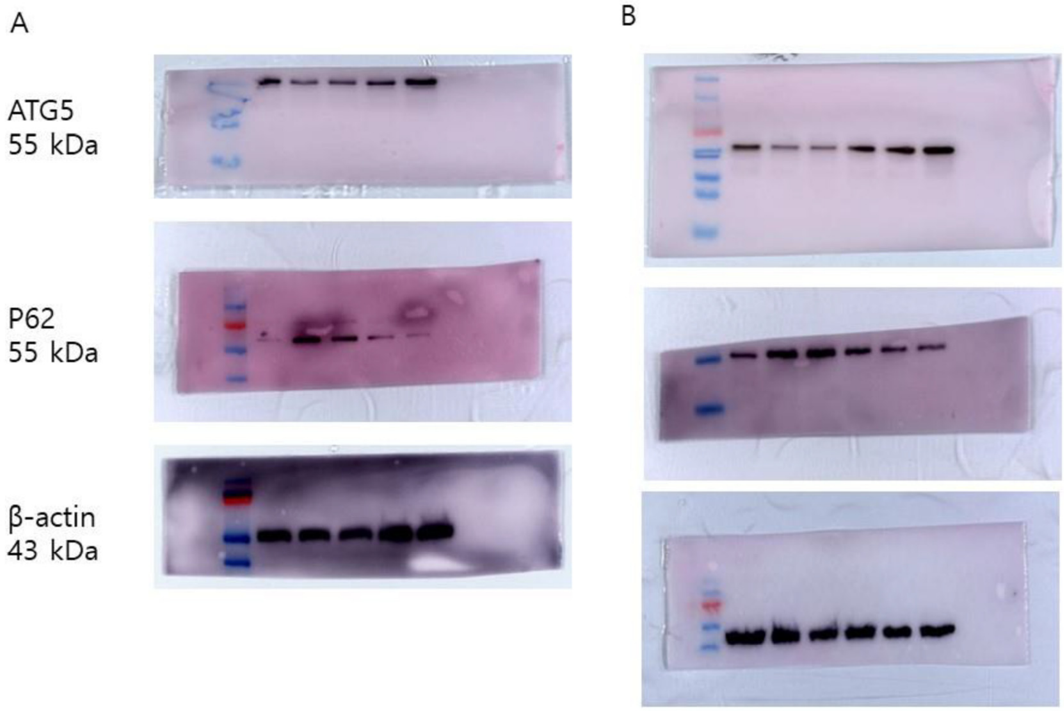

Fig. 6

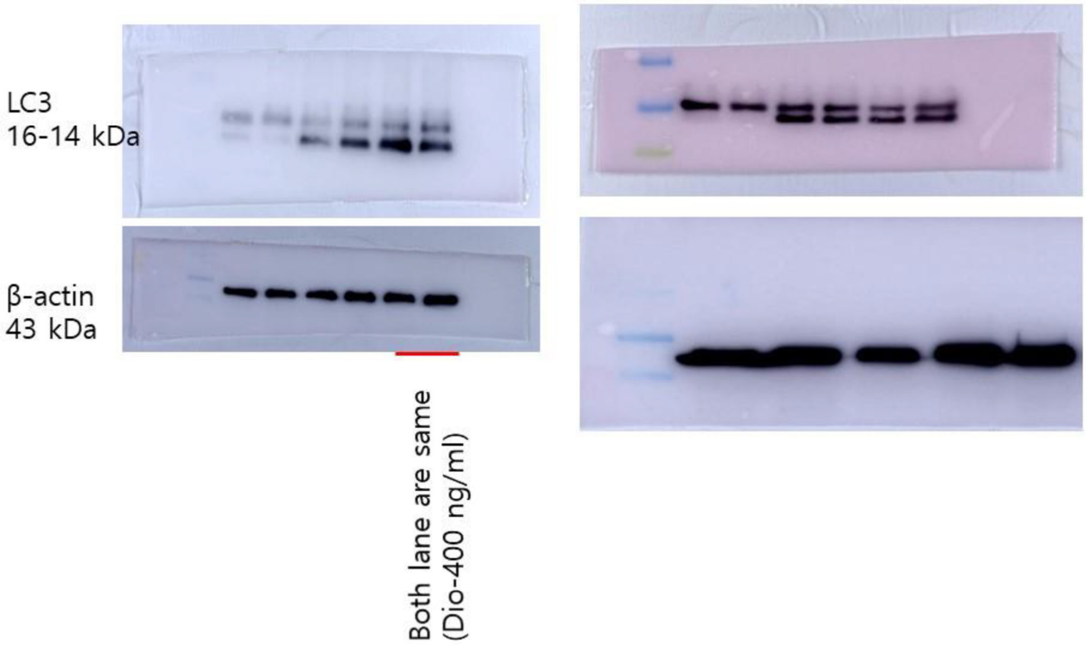

Supplement: Supplementary file 1 [file molecules-27-02827-s001.zip › molecules-1697414-supplementary.pdf]
